# Healthcare provider and health system leader perspectives on barriers to hypertension care in Malawi: insights from integrated and non-integrated HIV care settings

**DOI:** 10.1080/16549716.2025.2575569

**Published:** 2025-10-27

**Authors:** Christine Hagstrom, Pericles Kalande, Anu Aryal, Khumbo Phiri, Joep J. van Oosterhout, George Talama, Eric Lungu, Sam Phiri, Corrina Moucheraud, Risa Hoffman

**Affiliations:** a Department of Medicine, Division of Infectious Diseases, David Geffen School of Medicine, University of California, Los Angeles, Los Angeles, CA, USA; b Implementation Science Department, Partners in Hope, Lilongwe, Malawi; c Department of Health Policy and Management, Fielding School of Public Health, University of California, Los Angeles, CA, USA; d School of Global and Public Health, Kamuzu University of Health Sciences, Lilongwe, Malawi; e Department of Public Health Policy and Management, New York University School of Global Public Health, New York, NY, USA

**Keywords:** HIV, non-communicable diseases, integrated care, multimorbidity, Africa

## Abstract

**Background:**

Malawi has a significant burden of hypertension, including for people with HIV. The World Health Organization recommends integrated HIV-hypertension care, but such integration is not widely implemented in resource-constrained settings.

**Objective:**

This study explored barriers to hypertension care in Malawi from the perspectives of healthcare providers and health system leaders.

**Methods:**

We conducted a qualitative study of providers and health system leaders across 14 health facilities in Malawi. Interviews explored hypertension services in integrated (HIV and hypertension) and non-integrated clinics to identify barriers to hypertension care and compare barriers by integration status. Interview guides and analysis used the Consolidated Framework for Implementation Research. All transcripts were double coded and thematic analysis was performed.

**Results:**

From April–May 2023, we interviewed 33 individuals (25 providers and 8 health system leaders). Barriers to hypertension care were largely the same in integrated and non-integrated clinics and included stockouts of antihypertensive medications, lack of equipment, lack of provider training, and weak medical record systems. Providers working in integrated care emphasized the benefits of reduced burden for clients and improved quality of care but also reported unique challenges, including capacity constraints (due to the large number of clients) and inability to provide aligned dispensing of antihypertensive medications and ART (due to antihypertensive medication stockouts).

**Conclusions:**

Barriers to integrated HIV and hypertension care in our study largely reflected challenges for hypertension care more broadly. Future efforts should focus on provider training, supply chain strengthening, equipment procurement, and medical record system strengthening to improve outcomes for people with hypertension and hypertension-HIV multimorbidity.

## Paper Context

- **Main findings:** Healthcare providers and health system leaders in our study identified major barriers to hypertension care in Malawi, including medication stockouts, lack of equipment, insufficient provider training, and weak medical record systems, with similar challenges in both integrated HIV-hypertension care clinics and non-integrated clinics.
- **Added knowledge:** This study highlights the unique implementation challenges of providing hypertension care in resource-limited settings regardless of whether this care is delivered as part of HIV care or general care, emphasizing the need for improved antihypertensive medication supply chain and workforce training and capacity.
- **Global health impact for policy and action:** These insights can inform interventions to improve hypertension management in low-resource settings.

## Background

With advancements in care and treatment, HIV has become a manageable chronic condition, with many people having near normal life expectancy [1]. As a result, people with HIV (PWH) are facing an increased burden of non-communicable diseases (NCDs) associated with aging, including hypertension, diabetes, and cardiovascular diseases [2,3]. Low- and middle-income countries (LMICs), especially in southern Africa, have been particularly impacted by the increased burden of NCDs, with chronic diseases estimated to replace communicable diseases as the leading cause of mortality by 2030 [4,5]. Despite increasing burden of hypertension in southern Africa [6], hypertension diagnosis, treatment, and control remain low [7,8].

In Malawi, up to 30% of adults have hypertension [9] and an estimated 8.9% of adults are living with HIV [10]. National estimates of those with comorbid hypertension and HIV are limited, but estimates range from 24% to as high as 46% [11,12]. Care for HIV has traditionally been provided through antiretroviral therapy (ART) clinics, while care for hypertension is primarily provided in outpatient departments or NCD clinics, resulting in care fragmentation. Clients in Malawi with comorbid HIV and hypertension report greater barriers to accessing hypertension care as compared to HIV care, including difficulty accessing antihypertensive medications and increased cost of care [13].

The World Health Organization recommends leveraging HIV care infrastructure and integrating diabetes and hypertension care with HIV services for PWH [14]. Hypertension integration into existing HIV care infrastructure in African settings, using strategies such as unified medical record systems and task shifting, has improved blood pressure screening and client satisfaction; however, progress has been limited by medication supply chain issues, workforce shortages, and insufficient funding for scaling of integrated models [15–17]. There has been interest in scaling integrated care for HIV and hypertension (as well as other chronic diseases) in Malawi; however, to date, integration has been limited to a small number of facilities supported by non-governmental organizations, which have funding and human resources for implementation. We used qualitative methods to understand and compare barriers to hypertension care in Malawi from the perspective of healthcare providers working in integrated and non-integrated clinics, as well as health system leaders involved in management of HIV and NCD care programs.

## Methods

### Study setting

Malawi has a population of 18 million people [18]. As part of its essential health package, the Malawi government aims to provide free hypertension care at public sector health centers and district hospitals [19]. ART and HIV care are provided free of charge in all health centers. The Malawi Ministry of Health recommends that all PWH be screened for hypertension upon initiation of ART and annually thereafter [20]. While a small number of facilities in Malawi provide integrated care, most PWH requiring treatment for hypertension are referred to a separate NCD clinic within the facility or to a separate facility with the capacity for hypertension care.

This study was conducted under a parent study exploring client preferences for hypertension care, among adults both with and without HIV [21]. Both the parent study and this related substudy took place at 14 health facilities (10 government and 4 Christian Health Association of Malawi or ‘CHAM’ network) geographically spread across central and southern Malawi. All sites were supported by PEPFAR through implementing partners. Twelve clinics provided care and medications (including for hypertension) for free, and two clinics charged for hypertension care and medications. At the time of the study, six of the clinics provided integrated care for HIV and hypertension, defined as the same provider performing the clinical assessment and prescribing medications for both conditions during the same care encounter.

### Conceptual model and data analysis

We conducted semi-structured interviews using two tailored interview guides, one each for healthcare providers and health system leaders. The interview guides were informed by the Consolidated Framework for Implementation Research (CFIR), which theorizes that implementation can be affected by factors in the external or internal setting, the nature of the intervention, the attributes of the individuals involved, and the implementation process itself [22]. We focused on the domains relevant for low-income contexts, specifically on characteristics of systems (resource source and continuity; external funding priorities; and systems architecture) [23]. The provider guide focused on clinical practices and experiences with hypertension management, while the health system leader guide emphasized funding priorities, resource allocation, and policy considerations. Three members of the research team (RH, AA, and PK) developed the codebook deductively from the adapted CFIR constructs. AA and PK tested the codebook and identified and reconciled inconsistencies using three transcripts. All transcripts were then double coded using Atlas.ti 23 software (by AA and PK) and conflicts were resolved by a third-party tiebreaker (RH) if consensus could not be reached by the two coders. During coding, we added inductive codes for concepts that emerged outside the CFIR framework, resulting in a mixed approach that captured both anticipated and novel themes. We used summary statistics to describe participant characteristics. We performed thematic analysis of transcripts using Braun and Clark’s definition of themes [24]. We repeated this process until all codes were represented in themes and themes did not significantly overlap.

### Data collection

Eligible healthcare providers had at least one year of experience caring for clients in ART and/or NCD or outpatient clinics and key informants were eligible if serving in health leadership roles spanning HIV and NCD care, including from the Ministry of Health and district-level leadership/management positions. We purposively recruited two healthcare providers per facility, one from the ART clinic and one from the NCD/outpatient department clinic. When more than one provider was available, a single provider was randomly chosen based on availability for the interview. We selected other key informants based on our knowledge of individuals working in NCD and HIV care. The list was generated by study investigators and then edited based on feedback from leaders in the field working in Malawi. The sample size was determined to provide a sufficient number of participants to reach saturation of themes [25].

After verifying eligibility and obtaining oral informed consent, interviews were conducted in English (the primary language of healthcare providers and health system leaders) by experienced Malawian qualitative researchers (KP, PK, and EL). All interviews were audio recorded with permission of the respondent. On average, the interviews lasted 65 minutes (range 40–140 minutes). The audio recordings were transcribed and imported to Atlas.ti 23 for coding and analysis.

#### Ethical review

This study was reviewed and approved by the Ministry of Health, National Health Science Research Committee of Malawi (Number 20/07/2577) and the Institutional Review Board at the University of California Los Angeles (Number 20-001856).

## Results

We interviewed 33 individuals between April and May 2023. Twenty-five of these were healthcare providers, the majority of whom (*n* = 16, 64%) were clinicians (clinical officers and medical officers) or nurses (*n* = 8, 32%) (Table 1). The majority of healthcare providers delivered ART or hypertension care in non-integrated clinics (*n* = 19, 76%), while 6 (24%) were based in integrated clinics where hypertension and HIV care were provided by the same individual. Of the eight health system leaders, half were in management positions at district hospitals, two worked for the Ministry of Health supporting HIV and NCD programs, and two were in leadership positions of clinical programs focused on HIV and NCD care.

**Table 1.** Characteristics of participants (*n* = 33).

Healthcare provider characteristics	N = 25
Provider type n (%)	
Clinician (clinical officers, medical officers)	16 (64%)
Nurse	8 (32%)
Physician	1 (4%)
Median years of experience providing care, median (IQR)	
ART care	5 (8.5)
Hypertension care	7.5 (8)
Provider’s primary service area	
ART clinic	9 (36%)
NCD clinic or outpatient department	10 (40%)
Integrated ART-NCD clinic	6 (24%)
Facility characteristics	N = 14
Facility type n (%)	
District hospital	6 (43%)
Rural hospital	2 (14%)
Health center	1 (7%)
Mission hospital	4 (29%)
Central Hospital	1 (7%)
Facility region n (%)	
Central	10 (71%)
Southern	4 (29%)
Facility ownership n (%)	
Christian Health Association of Malawi	4 (29%)
Government	10 (71%)
Non-provider, health leadership key informants	N = 8
Type of key informant n (%)	
Ministry of Health leader engaged in HIV and/or NCD care	2 (25%)
Leadership role in clinical program for HIV/NCD care	2 (25%)
District hospital managers	4 (50%)

## Barriers to hypertension care

### Frequent antihypertensive medication stock outs

Most providers cited antihypertensive medication drug supply as a significant barrier to providing effective hypertension care for all clients, regardless of clinic integration status. Several providers described needing to adjust clients’ hypertension treatment as a result of a medication stockout, or referring clients to another facility with a better antihypertensive medication supply.

As a small hospital, we have a limitation in terms of drug supply. We always refer our clients to [another clinic] to get other necessary drugs because we do not keep all those in stock‬. (HIV care provider, non-integrated clinic)‬‬

Sometimes on the issues of drugs, you find out that we do not have some drugs for over six months … and we are always forced to switch their drug treatment to other drugs. (HTN care provider, non-integrated clinic)

Many providers reported that sending clients to buy antihypertensive medication at other facilities leads them to return to follow-up visits without medications, and several providers connected this to an ultimate lack of blood pressure control.

Oftentimes we have regular drug stockouts. So, you see, you have prescribed drugs for a client today thinking that he will get them, but the drugs are not there, even if you ask them to buy, they will not buy. They will come back, they still have spiraling BPs [blood pressures]. You would think that you are not managing them yet they are not getting the drugs so those are some of the challenges and they end up developing complications like cerebrovascular accidents. (HTN care provider, non-integrated clinic)

Health system leader key informants echoed concerns about antihypertensive medication stockouts. They also cited the challenge of limited budgets for medications, which require facilities to prioritize how to divide funds across health programs – with priority often given for more urgent health issues, such as infectious diseases, maternity care, and surgical problems.

Central medical stores supply is erratic and we prioritize … We prioritize maternity, theater [operating room], etc. And then chronic disease is not part of that, like it’s not seen as something that we need to prioritize whenever we’re procuring drugs.‬ (Health system leader)‬‬‬‬‬‬‬

Antihypertensive medication shortages pose a unique challenge for HIV-hypertension care integration. Providers expressed wanting to align multi-month dispensing of ART and antihypertensives to reduce the burden of care on clients who are stable (controlled for both diseases) but were unable to do so due to insufficient antihypertensive medication supply.

We have challenges in that regard because out of the clients that we have at ART, very few collect ART on a monthly basis. Most of them collect 6 months supply and the least collect 3 months supply. But if you look at NCD [antihypertensive medication], it is monthly supply. So, we try to harmonize the visits. If we can have adequate supply then we can be well harmonizing with ART supply. If ART is 6 months then we can do the same at NCD [for antihypertensive medication]. (HIV care provider, non-integrated clinic)‬

### Lack of functional equipment for hypertension care

Healthcare providers in both integrated and non-integrated care settings reported lack of necessary equipment as a frequent barrier to providing effective hypertension care.

Our machines, we always have challenges, sometimes you will hear that the BP machine has no batteries, the weighing scale is not functioning. (HTN care provider, non-integrated clinic)‬‬

As a facility we get support from DHO [District Health Office], so sometimes because of financial issues they are unable to provide us with the batteries that we use in BP machines … And some of the BP machines that are being procured are substandard, they work only for a month and the next month you find that it’s not functional.‬ (HIV care provider, non-integrated clinic)‬‬‬

Our BP cuff is malfunctioning and I need to borrow from another department but when I find that is in use there, we have our clinic without BP cuff.‬ (HTN care provider, non-integrated clinic)‬‬‬

Health system leaders also raised concerns about equipment as a major barrier to hypertension care, citing issues with lack of functional equipment and challenges with security to keep items safe.

Often they do not have functioning blood pressure machines to use … They will always say we do not have batteries for that machine so therefore we can’t do blood pressure so I think regular testing of blood pressure and all clinical settings obviously need to be re-enhanced‬‬. (Health system leader)‬‬‬‬‬‬‬‬‬‬‬‬‬‬‬‬‬‬‬‬‬‬‬‬‬‬‬‬‬‬‬‬‬‬‬‬‬‬‬‬‬‬‬‬‬‬‬‬‬‬‬

… a challenge comes in not because the equipment is not there, the equipment is there but these digital ones do get broken easily, they don’t last long. Sometimes there may be issues of theft and the challenge with our infrastructure is that we do not have a fence so it becomes very hard for us to trace who are stealing these machines.‬ (Health system leader)‬‬‬‬‬‬

### Weaknesses in medical records for hypertension care

Almost all participants described challenges with medical records related to hypertension care. This was true for providers serving clients with only hypertension as well as those with HIV and hypertension. They described haphazard documentation, or in some cases, solely documentation in the client’s personal health record, which is a small booklet (called a ‘health passport’) carried by Malawians that contains limited documentation. This book can be easily lost or forgotten and therefore not available for the clinician to review.

When a client is referred here, they only show us the health passport and in it there is no history as regards to what was prescribed or any impact of a particular drug, so as a health facility we are supposed to have such information, we cannot be relying on a health passport – what if they bring a new one each week? If we cannot trace their history we may end up providing a wrong prescription especially if they meet a different doctor. (HTN care provider, non-integrated clinic)

… the majority [of records] are paper based and personal held record [health passport] so it’s very hard to ensure continuity of care particularly for people moving from one facility to another so it’s good to have centralized electronic medical record systems, which would make a huge difference for management for long term conditions.‬ (Health system leader)‬‬

With regard to individuals with HIV and hypertension cared for in non-integrated clinics, several providers reported not having any information about a client’s hypertension care and therefore not being able to co-manage or support management of this condition.

The tricky part is that these people use different books … They use a health passport for ART services and another health passport for NCD clinic, so it is difficult to flag them out that this particular client is in both clinics. So, if s/he comes with only the ART profile we cannot track as a result, we just manage them the same way as we manage the ordinary [ART] clients‬. (HIV care provider, non-integrated clinic)

Similarly, most ART providers in non-integrated clinics expressed frustration in not knowing the outcome when referring clients to NCD clinic for hypertension care and not being able to easily communicate with NCD providers to coordinate care and optimize treatment.

I think this is affecting linkage because what it means is that a client who is on ART and is a new client for NCD mostly we just refer them to go to NCD clinic we don’t even follow up if the person has really gone there, or has the client gotten the management or treatment‬. (HIV care provider, non-integrated clinic)

### Limited provider capacity for hypertension care

Most providers stated that they had received very little, if any, continuing professional development or refresher training to provide hypertension care since their initial medical training. This was the case for providers in both integrated and non-integrated clinics.

I have not received any kind of training. We are using the knowledge we acquired from school … This is science, and it’s dynamic. It changes day in and day out. So, there is need [to] have a number of trainings. Even the mentorship programs should be there. (HTN care provider, non-integrated clinic)‬‬‬‬‬‬‬‬‬‬‬‬‬‬‬‬‬‬‬‬‬‬‬‬‬‬‬‬‬‬‬‬‬‬‬‬‬‬‬‬‬‬‬‬‬‬‬‬

Even a layman knows there is hypertension but then not every health provider knows how to go about caring for these hypertensive clients. There are a lot of gaps, starting from diagnosis, going to monitoring and choice of medication. Being able to know that once a client has reached this level, we need to give them this medication or we need to start them with this medication. Not every health provider out there knows this.‬‬ (HIV and HTN care provider, integrated clinic)

Gaps in providers’ knowledge can result in poor quality of care and failure to achieve blood pressure control.

Sometimes the problems can come from providers, for example, some people don’t know how to interpret the readings. A client can come with high BP in high ranges but the provider will just look at the dates and provide meds [refills] … . If the client doesn’t ask then they might go home with high BP and then develop stroke … So if the provider isn’t knowledgeable, that can accelerate many clients developing BP complications that we could have controlled here initially.‬ (HIV care provider, non-integrated clinic)‬‬

Health system leaders also emphasized the importance of training in both ART and NCD care, so that care providers are able to manage both conditions within integrated clinical contexts.

So, our plan and our vision is integration of HIV and NCDs. All ART providers, we are speaking of cadres that are able to prescribe medication, these should be clinicians or nurses. They should be trained to manage NCDs and that all NCD provider, particularly those that are able to prescribe medication, in this case clinicians and nurses in NCD care, should also be trained in HIV care. (Health system leader)

Further complicating lack of knowledge and skill around hypertension care, ART providers and health system leaders also raised concerns that there are not enough care providers (overall, or not in the right cadre) to cover client care needs in an average ART clinic day, making the addition of hypertension care to the already-heavy workload extremely challenging.

Sometimes even here when there is only one care provider and there is too much work, they just focus on finishing the waiting line [for ART], not necessarily checking if a person has had their BP checked as well as the interpretations.‬ (HIV care provider, non-integrated clinic)

The issue of skills is very crucial and also, we look at the caliber but also nature of staff, human resource that we have in our facilities. For example, at health centers, where we have a medical assistant but not a clinical technician, not a clinical officer, not a medical officer. So these health centers are the ones that would probably need a lot more support. (Health system leader)

Overall, barriers were largely consistent across integrated and non-integrated settings, including frequent medication stockouts, lack of functional equipment, limited provider training, and weak medical record systems. Minor differences emerged: providers in integrated clinics more often described challenges aligning dispensing intervals of ART and antihypertensive medications, while those in non-integrated clinics more frequently reported difficulties coordinating care for ART clients receiving hypertension care at other facilities.

## Benefits of integration

While most study participants focused on barriers to the provision of hypertension care, several positive aspects of integration were noted, particularly from providers with experience working in these settings.

### Ability to provide holistic care

All integrated care providers emphasized that this allows for the provision of a more comprehensive, holistic approach to clinical care. One provider explained:

Full integration is the best because when you treat someone there are a lot of things that come into the care provider’s mind. An example would be switching the drugs. Sometimes a hypertensive person can have four drugs and if they are only to go to the NCD clinic, NCD will only focus on their part. The moment they come for the ART the provider would also give him more tablets and you will see that the client will be taking more than five tablets a day. By the end of the day the client will choose to take either ART or hypertensive drugs. But integrating can actually help the provider to make a better judgement before the drugs are provided to the client.‬ ‬(HIV and HTN care provider, integrated clinic)

Providers with experience in integrated care also noted the benefit of being able to monitor for side effects – and this also facilitates clear communication with the client.

Sometimes it happens that this client is getting ART drugs and he is developing some side effects and some medication for hypertension that also cause the same side effects. If that client goes to one particular person for HIV and hypertension, I think that’s way better because monitoring of side effects will be done effectively. If it happens that a client comes here with issues and then we tell him ‘The side effects are for these drugs.’ But if they go somewhere else they will be told ‘No, the side effects are not these drugs,’ in such a way will confuse the client.‬‬ (HIV and HTN care provider, integrated clinic)

### Reduced client burden and increased client satisfaction

Most providers working in integrated HIV and hypertension clinical settings also noted a benefit for clients related to reducing the burden of care from travel and opportunity costs that result from fragmented care.

… we have people who come from afar, with full integration, they will not spend a lot of time, or a lot of money on transport since they will get all the help, they need at one point and I think it is very possible in Malawi, we can do it.‬ (HIV and HTN care provider, integrated clinic)‬

Health leaders agreed that integrated care models alleviate the burden that clients with multimorbidity face when seeking care.

Most of the clients take so much time when they have come for care in the facilities. So, what we want is to reduce the time that they are spending in the hospitals because if someone goes to collect their ARVs, there is already a queue at the hypertension clinic. When they are coming from there, they will find a long queue. It means they will stay the whole day and some of them are diabetic. It means they came without food … So, what we want is, if they are HIV positive and hypertensive, they would never come to the hypertension clinic. They would be taken care of at the ART clinic. So it is like comprehensive care; whether they are diabetic, they have other problems, depression they would be sorted there in the ART clinic. (Health system leader)

Finally, most providers currently working in an integrated clinic had prior experience working in more fragmented care contexts. These individuals perceived that integrated care has improved health outcomes and clients’ experiences coming to the clinic:

Aaaaah, this is not to boast but we are doing very fine because you can even hear from the clients giving feedback. Like I said previously, before we used to tell them to go get the drugs somewhere else or go and buy. Now since we started providing the medication right here, the clients are happy. We have a quite large number of clients, who previously used to have high BP but now they are controlled because they are able to get medication here … Even if it was me, I wouldn’t prefer going to 2 different facilities in a single day.‬‬ (HIV and HTN care provider, integrated clinic)

## Discussion

Our qualitative data suggest that the barriers to HIV and hypertension care within integrated clinics in Malawi stem from challenges in the health system that impact care for all people with hypertension, regardless of HIV status. Lack of functioning equipment, such as blood pressure cuffs, has been documented as a barrier to hypertension care in similar contexts [26,27]. In addition to equipment, one of the most critical bottlenecks identified in our interviews was the availability of antihypertensive medications. NCD medication availability is a widespread problem in low- and middle-income countries, including in Africa, largely due to inadequate funding for medications and human resources to support supply chain management. Financial support for the NCD supply chain is currently sparse and may dwindle further as PEPFAR programs are scaled down across Africa [28,29], placing more strain on resources for health in high prevalence HIV contexts like Malawi. Given that all facilities in our study received PEPFAR support, a reduction in funding could have a substantial negative impact on integration by limiting resources (including staff, supplies, and medications). Our study focused on integrating hypertension care within ART programs, reflecting the current structure of HIV service delivery in Malawi, and we did not address approaches that integrate HIV and NCD services into primary health clinics. As PEPFAR support declines, models of fully integrated primary health care in which people with HIV and NCD co-morbidities are managed alongside adults without HIV may become increasingly important. This is an important area for future research.

Strengthening supply chain management approaches may help reduce scarcity in resource-constrained African settings. Malawi primarily uses a multi-tiered distribution system in which essential medications are distributed from central warehouses to regional warehouses to health facilities [30]. Moving towards a more direct distribution system may improve the efficiency of the supply chain. A study in Zambia found that a direct distribution system in which clinics order and receive medications directly from a central agency significantly reduced the duration and frequency of stockouts compared to a multi-tiered distribution system [31]. Further, there are over 20 non-integrated information systems in Malawi for supply chain operations, including ordering, warehousing, and transportation. The lack of integrated systems creates challenges for tracking inventory and responding promptly to stockouts [30]. Digitizing and integrating information systems to manage all aspects of the supply chain in Malawi could improve inventory management and reduce stockouts [32,33]. Successful implementation of these approaches would require substantial policy changes and financial resources.

Similar to previous studies in Africa [34,35], we identified understaffing as a key barrier to providing hypertension care. The availability of providers is particularly challenging in ART clinics in Malawi, where a single provider may see over 50 clients per day and thus cannot easily integrate hypertension care into their already heavy workload. Research suggests that task shifting, where responsibilities are delegated to lower-level cadre workers (including lay staff), may be a successful strategy to address capacity issues and improve client outcomes [36,37]. For example, in Ghana, nurse task shifting of hypertension care, including cardiovascular risk assessment, lifestyle counseling, and initiation/adjustment of antihypertensive medications, was associated with a significant increase in blood pressure control among clients with uncontrolled hypertension [38]; however, implementation of task-shifting requires significant support. A study in Nigeria found low facility readiness and resources to implement task-shifting for hypertension management of PWH [39]. Task-shifting is a potential strategy to increase capacity for hypertension in Malawi but would require careful planning to ensure other health services are not compromised.

We also found lack of provider knowledge and skills to be a barrier to hypertension care in Malawi. As with other barriers, this was relevant for both integrated and non-integrated care. Almost all providers in our study requested refresher training in hypertension care. This highlights the need for continued education and support, as evidenced by a recent study in Malawi that found training significantly improved mid-level providers’ knowledge of NCD care in an integrated clinic – and ongoing clinical mentorship improved provider confidence, particularly in treating complicated cases [40]. Communities of practice, or ‘a group of individuals who share a common interest or passion and engage in a collective process of learning over time’ [41] may also be a successful strategy to enhance hypertension care at low cost by facilitating an environment that supports improving provider knowledge and skills and fostering collaboration between providers within and across facilities. There are promising data from Malawi on the use of WhatsApp to develop communities of practice across borders. This strategy was successfully used to connect subspecialists in the United States (US) with Malawian clinicians and trainees (from the US and Malawi), to discuss challenging clinical cases. An evaluation found improved patient outcomes and improved knowledge among participants [42]; however, acceptability may be an issue, as utilization was low, particularly among Malawian (compared to US) clinicians.

We found that challenges with medical records for hypertension care negatively impacted providers’ ability to provide holistic care, and this issue was particularly salient in the integrated care contexts for ART providers who felt reluctant or unable to provide hypertension care due to lack of available information about their clients’ antihypertensive medications. Adoption of electronic medical record (EMR) systems is limited across low-resource settings, largely due to insufficient funding, infrastructure, and lack of provider training [43]. While Malawi does have a point-of-care EMR system at high-burden ART clinics that includes a specific module for hypertension screening and management, its utilization has been limited to date, likely due to challenges faced with EMR systems in similar settings such as cost of maintenance and lack of prioritization and training [43]. Training and mentoring staff to use EMR and utilizing focal persons (‘champions’) at health facilities for integrated hypertension-HIV data collection may improve utilization in Malawi. A study in Tanzania found that ease of use, training, and including staff in the development of EMR systems and protocols enabled successful implementation [44]. However, successful implementation also depends on addressing infrastructure challenges. For example, electricity is a barrier to continuous use of EMR systems in Malawi due to the fragile power grid. A study of providers in Ghana found that while they perceived EMR systems as beneficial for client care and workflow, they expressed frustration when erratic power supply prevented them from using the system [45].

Importantly, providers in our study with experience working in clinics providing integrated hypertension and HIV care had a positive view of integration. They reported that it allowed them to deliver higher quality care and reduced burdens on clients. Although we did not interview clients, a scoping review of client perspectives on integrated care for HIV, hypertension, and diabetes similarly found that clients viewed integrated care positively, highlighting reduced stigma, reduced opportunity costs, and an appreciation for more holistic, client-centered care as key benefits compared to fragmented care [46]. Integration has shown to improve staff capacity and client retention [47].

## Limitations

The sample of health facilities that participated in our study was small and all were supported by PEPFAR implementing partners; our findings may therefore not fully capture the experiences and perspectives of providers across Malawi who face varied barriers to providing hypertension care. Second, only a minority of participants had direct experience providing integrated hypertension and HIV care, potentially limiting our ability to represent the full range of challenges and opportunities associated with integrated care. Third, our study did not include clients, and further research should engage with recipients of care to better understand their preferences and experiences with managing hypertension.

## Conclusions

Our findings highlight that many of the barriers to providing integrated HIV-hypertension care in Malawi reflect general challenges in providing hypertension care. Strengthening the health system for NCD care, including a focus on improving the supply chain, ensuring availability of functional equipment, offering provider training, and improving medical recordkeeping, will be crucial to successfully providing hypertension care across the spectrum of clients, including those living with HIV and hypertension comorbidity. More studies are needed to understand how to address barriers to hypertension care (including integrated care) with a focus on cost-effectiveness and sustainability in resource-constrained settings.
